# NSD Family-Mediated H3K36 Methylation in Human Cancer: Mechanisms and Therapeutic Opportunities

**DOI:** 10.3390/biomedicines13112749

**Published:** 2025-11-11

**Authors:** Jae Eun Park, Minh Tuan Nguyen, Jaehee Kim, Chang Hoon Lee, Jin-Wu Nam, Heekyoung Chung, Mi Kyung Park, Jeong-Yeon Lee

**Affiliations:** 1 Department of Pathology, College of Medicine, Hanyang University, Seoul 04763, Republic of Korea; silver9336@gmail.com; 2 Pharmaceutical Biochemistry, College of Pharmacy, Dongguk University, Goyang 10326, Republic of Korea; nmtuan28@dgu.ac.kr (M.T.N.); uatheone@dongguk.edu (C.H.L.); 3 Department of Biomedical Science, Hwasung Medi-Science University, Hwaseong 18274, Republic of Korea; jhkim@hdadmbio.com; 4 Department of Life Science, College of Natural Sciences, Hanyang University, Seoul 04763, Republic of Korea; jwnam@hanyang.ac.kr; 5 Hanyang Institute of Advanced BioConvergence, Hanyang University, Seoul 04763, Republic of Korea; 6 Hanyang Institute of Bioscience and Biotechnology (HY-IBB), Hanyang University, Seoul 04763, Republic of Korea

**Keywords:** cancer, epigenetics, histone H3 lysine 36, histone methylation, NSD family

## Abstract

Histone H3 lysine 36 (H3K36) methylation, a pivotal epigenetic mark that ensures transcriptional fidelity and genomic integrity, plays an essential role in development and tumorigenesis. The nuclear receptor-binding SET domain (NSD) family of histone methyltransferases, comprising NSD1, NSD2, and NSD3, primarily catalyzes mono- and di-methylation of H3K36 (H3K36me1/2) and engages with chromatin-associated and transcriptional regulatory complexes in a context-dependent manner. Increasing evidence demonstrates that NSD family members have emerged as critical drivers in human cancers. Recurrent gene amplifications, point mutations, and oncogenic fusions of NSD family genes are frequently observed in both solid and hematologic cancers. Their dysregulation contributes to tumorigenesis, cancer cell proliferation and survival, and metastatic progression through both H3K36 methylation-dependent and -independent mechanisms. Pharmacological inhibition of NSD catalytic activity, as well as alternative approaches such as targeted protein degradation or disruption of cofactor interactions, are emerging as promising therapeutic strategies for cancer treatment. This review summarizes the structural features, molecular functions, and cancer-associated alterations and mechanisms of the NSD family and highlights recent advances in targeting these enzymes as potential epigenetic vulnerabilities in cancer.

## 1. Introduction

Epigenetics refers to heritable changes in gene expression without altering the underlying DNA sequences. Unlike genetic mutations, epigenetic modifications are reversible and dynamically regulated in response to various environmental stimuli and cellular processes. Epigenetic regulation, including DNA methylation, histone modifications, and chromatin remodeling, plays a pivotal role in orchestrating gene expression programs that govern cell identity, differentiation, and homeostasis [1,2]. Among these modifications, histone methylation serves as a highly specific and finely tuned mechanism of transcriptional regulation [3,4,5]. Depending on the methylation site and degree (mono-, di-, or tri-methylation), it can either activate or repress gene expression. One of the most functionally significant histone marks is methylation of lysine (K) 36 on histone H3 (H3K36), which influences chromatin accessibility, transcriptional elongation, and long-range DNA methylation [6]. The nuclear receptor-binding SET domain (NSD) family of histone methyltransferases, comprising NSD1 (KMT3B), NSD2 (MMSET/WHSC1), and NSD3 (WHSC1L1), plays a critical role in catalyzing mono- and di-methylation of H3K36 (H3K36me1/2). These enzymes are essential for normal development, chromatin organization, and transcriptional regulation [7,8,9].

Epigenetic dysregulation is a fundamental hallmark of cancer that drives tumor initiation and progression by controlling oncogenic transcriptional programs [10,11]. Aberrant H3K36 methylation mediated by NSD family members has been implicated in diverse human malignancies [12,13]. Across multiple tumor types, NSD family members are frequently altered by genetic events, such as amplification, chromosomal translocation, and point mutations, and their overactivity affects the chromatin landscape, reprograms transcription, and promotes tumor initiation and progression [7,8,9]. For instance, the NUP98-NSD1 fusion resulting from chromosomal translocation in acute myeloid leukemia (AML) aberrantly induces H3K36me2 and contribute to leukemogenesis [14,15,16,17]. In multiple myeloma, NSD2 overexpression caused by the t(4;14) translocation represents a key oncogenic event. Furthermore, gain-of-function mutations in *NSD2* in acute lymphoblastic leukemia (ALL) and several solid tumors enhances methyltransferase activity, thereby driving tumorigenesis [18,19,20,21]. *NSD3* gene is recurrently amplified at chromosome 8p11.23 in several solid tumors, particularly in breast cancer and lung squamous cell carcinoma, and has recently gained attention as a potent oncogenic driver that promotes tumorigenesis and metastatic progression [22,23,24,25]. In addition, short NSD isoforms lacking the catalytic SET domain contribute to tumorigenesis via interactions with various epigenetic and transcriptional regulators [26,27]. Overall, NSD family can reshape the epigenetic and transcriptional landscapes of tumor cells in both H3K36 methylation-dependent and –independent manners, thereby contributing to various malignant phenotypes, including tumorigenesis, cancer cell proliferation, epithelial–mesenchymal transition (EMT), invasion and metastasis, as well as therapeutic response, highlighting NSD1, NSD2, and NSD3 as promising epigenetic therapeutic targets.

This review comprehensively summarizes the general functions of NSD family members in modulating epigenetic gene expression, alterations in *NSD1/2/3* genes across multiple human cancers, H3K36 methylation-dependent and -independent mechanisms of NSD family genes in modulating diverse malignant phenotypes, and potential therapeutic strategies for targeting NSD family in human cancers.

## 2. H3K36 Methylation and NSD Family Methyltransferases

### 2.1. Roles and Regulators of H3K36 Methylation

Histone modifications represent a fundamental mechanism of epigenetic gene regulation. Various post-translational modifications occur on histone tails, including acetylation, methylation, phosphorylation, and ubiquitination, each exerting distinct effects on chromatin structure and transcription factor accessibility [3,4]. Among these, histone methylation is considered one of the most sophisticated and stable forms of transcriptional control, enabling precise regulation of gene expression that underlies cell-fate decisions and normal development [5]. Histone methylation primarily occurs on lysine (K) or arginine (R) residues of histone proteins and can exist in mono-, di-, or tri-methylation states. Depending on the residue and degree of methylation, these marks can either activate or repress transcription. For instance, methylation of H3K4, H3K36, H3K79, and H4K20 are often associated with active gene transcription, whereas H3K9 and H3K27 methylation are linked to transcriptional repression. These marks are dynamically regulated by histone methyltransferases (writers) and histone demethylases (erasers), and are recognized by methyl-lysine binding effectors (readers) to orchestrate transcriptional programs [5,28].

H3K36 methylation is a central epigenetic modification that governs proper gene transcription and maintains genomic stability [6]. The distinct methylation states of H3K36 confer specialized regulatory outcomes: H3K36me1 is broadly distributed across the genome and is often regarded as an intermediate state with limited transcriptional impact [29]. By contrast, H3K36me2 and H3K36me3 are functionally linked to transcriptional activation. H3K36me2, the most abundant form of H3K36 methylation, is enriched in intergenic and regulatory regions, whereas H3K36me3 is predominantly found within gene bodies [30,31,32]. The degree of H3K36 methylation is tightly regulated by site-specific enzymes. H3K36me1 and/or H3K36me2 are mainly catalyzed by the NSD family, as well as additional methyltransferases including ASH1L (ASH1-like), SETMAR (SET domain and mariner transposase fusion gene-containing), SMYD2 (SET and MYND domain-containing 2), and SETD3 [6,33]. Meanwhile, SETD2 is solely responsible for H3K36me3 [34,35]. The methylated H3K36 can be removed by Jumonji C (JmjC) domain-containing family of histone demethylases (JHDMs), including JHDM1 and JHDM3 for demethylation of H3K36me1/2 and H3K36me2/3, respectively [28,36]. Several proteins containing PWWP (proline-tryptophan-tryptophan-proline), tudor, and chromodomains, act as readers for H3K36 methylation marks to orchestrate transcriptional regulation. Collectively, this coordinated network of H3K36 methylation writers, erasers, and readers plays a fundamental role in maintaining transcriptional homeostasis.

### 2.2. NSD Family Members, Structures, and Isoforms

**Figure 1:**
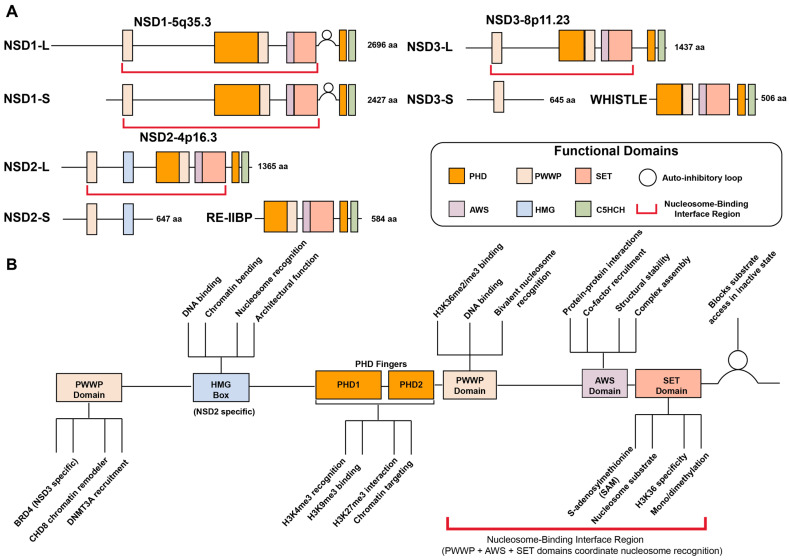
Domain architecture and functional organization of NSD family methyltransferases. (**A**) Schematic representation of the conserved domain structure of long (L) and short (S) isoforms of NSD1 (chromosome 5q35.3), NSD2/MMSET/WHSC (chromosome 4p16.3), and NSD3/WHSC1L1 (chromosome 8q11.23). All family members contain PHD finger domains (PHD1 and PHD2), a PWWP domain, and the catalytic SET domain flanked by pre-SET and post-SET regions. NSD2 uniquely contains an HMG box domain. The autoinhibitory loop is located between the SET domain and C-terminal region. The nucleosome-binding interface region (indicated by blue brackets) spans multiple domains. Amino acid lengths for each domain are indicated. (**B**) Functional roles of individual domains within NSD proteins. PHD fingers mediate protein–protein interactions, histone mark recognition (H3K4me3 via PHD1, H3K9me3 via PHD2), architectural function, and chromatin targeting. The PWWP domain functions as a chromatin reader, recognizing H3K36me2/3 marks and facilitating recruitment of cofactors including BRD4 (NSD3-specific), CHD8 chromatin remodeler, and DNMT3A for DNA methylation. The HMG box (NSD2-specific) contributes to DNA binding, chromatin binding, nucleosome recognition, and architectural function. The catalytic SET domain, along with pre-SET and post-SET regions, catalyzes H3K36me1/2 using SAM as a methyl donor. The autoinhibitory loop regulates SET domain activity by modulating substrate access and catalytic efficiency. The nucleosome-binding interface region, comprising coordinated interactions between PWWP, AWS, and SET domains, is essential for nucleosome recognition, proper substrate positioning, H3K36-specific methyltransferase activity, complex assembly, and structural stability of the catalytic core.

The NSD family of histone methyltransferases comprises three members: NSD1, NSD2 (also known as MMSET or WHSC1), and NSD3 (WHSC1L1) [14,25,37]. These highly homologous enzymes share conserved domain structures, including a catalytic SET (su(var)3–9, enhancer of zeste and trithorax) domain, PWWP domains, and multiple PHD (plant homeodomain) zinc finger motifs, which collectively coordinate enzymatic activity, chromatin binding, and protein–protein interactions (Figure 1).

The SET domain serves as the core catalytic unit of NSD proteins, transferring a methyl group from S-adenosylmethionine (SAM) to H3K36. Structurally, this domain is flanked by pre-SET and post-SET subdomains that stabilize the catalytic core and ensure proper substrate recognition, both of which are essential for enzymatic function [7,38,39,40,41]. A distinctive structural feature of NSD proteins is the presence of an autoinhibitory post-SET loop that connects the SET and post-SET domains. In the crystal structure of NSD1, this regulatory loop adopts a closed conformation that physically blocks the substrate lysine-binding channel, preventing access of H3K36 to the bound SAM and thereby maintaining the enzyme in an inactive state. Molecular dynamics simulations and computational docking studies reveal that this loop can undergo conformational changes to adopt an open, active conformation, particularly upon interaction with nucleosomal DNA, which stabilizes the active state and allows H3K36 to access the catalytic site. This autoinhibitory mechanism provides an elegant explanation for the strong nucleosomal substrate selectivity exhibited by NSD proteins and represents a novel regulatory strategy among SET domain histone methyltransferases [38]. In certain context, the SET domain may also target non-histone substrates, expanding the functional diversity of NSD enzymes [42,43,44,45,46].

The PWWP and PHD zinc finger domains contribute to both substrate targeting and methyl-mark recognition, enabling NSD proteins to function as both writers and readers of H3K36 methylation. The PWWP domain acts as a reader module that specifically recognizes H3K36me2 and H3K36me3 marks, anchoring the NSD proteins to chromatin and facilitating interactions with transcriptional regulators such as BRD4 and CHD8 [26,47,48]. The PHD finger domains also function as histone readers by recognizing various histone modifications including H3K4me3, H3K9me3, and H3K27me3, enabling precise genomic targeting and fine-tuning of gene expression [48,49,50,51,52,53]. The HMG (high-mobility-group) box, which is uniquely present in NSD2, supports non-sequence-specific DNA recognition or interaction with other DNA-binding factors [54,55,56].

The nucleosome-binding interface region encompasses the PWWP domain (H3K36me2 reader) [48], AWS-SET-post-SET catalytic core, N-terminal loop, and PHD domains [57]. These elements coordinate to recognize nucleosomal substrates through DNA unwrapping (~20 bp), multi-domain chromatin engagement, and relief of autoinhibition via the post-SET loop conformational change [57]. This architecture enables specific H3K36 methylation and explains the nucleosome-dependency of NSD enzymatic activity.

Isoform diversity further expands the functional complexity of the NSD family. Each member exists in multiple alternative spliced forms, with distinct domain compositions and biological functions [7,8]. NSD1 expresses both a full-length long isoform (NSD1L; 2696 amino acids) and shorter variants (NSD1S; 2427 amino acids) that nonetheless retain the major functional domains, and consequently, the differences in function between these isoforms are thought to be minimal [58,59,60]. NSD2 is expressed as three major isoforms: the catalytically active full-length isoform MMSET II (NSD2L; 1365 amino acids) responsible for H3K36me1/2; the shorter MMSET I isoform (NSD2S; 647 amino acids), which lacks the SET domain [37,61]; and the RE-IIBP isoform (584 amino acids), which retains the catalytic domain but exhibits altered substrate specificity or distinct functions, including RNA splicing and pre-rRNA processing [62,63,64,65]. Similarly, NSD3 is also characterized by three main isoforms: the full-length long form (NSD3L; 1437 amino acids) possesses a functional SET domain catalyzing H3K36me2 [25,66]; the short form (NSD3S; 645 amino acids) lacks the catalytic SET domain [26]; and the NSD3-Whistle isoform (506 amino acids), an unusual, truncated variant generated by alternative splicing or chromosomal rearrangements, retaining SET and PWWP domains but with poorly understood functions [67]. While the shorter isoforms of NSD2 and NSD3, which lack the SET domain, have no methyltransferase activity, they play key roles in modulating gene expression by interacting with chromatin remodelers or other transcriptional regulatory complexes, thereby functioning as transcriptional adaptors [26,27]. Together, the diversity of isoforms and domain architectures within the NSD family enables a complex regulatory capacity to modulate chromatin states and gene expression programs.

### 2.3. Biological Functions of NSD Family

#### 2.3.1. Epigenetic Regulation of Gene Expression

The NSD family members play a central role in modulating gene expression via epigenetic mechanisms. As key histone methyltransferases containing a conserved SET domain, NSD family enzymes are primarily responsible for catalyzing H3K36me1/2 to activate gene transcription. While an early report indicated that NSD1 could methylate both H3K36 and H4K20 [68], subsequent studies have highlighted its strong substrate preference for H3K36me2 [38,40]. NSD1-mediated H3K36 methylation also facilitates RNA polymerase II (RNAP II) recruitment at promoter regions and its transition from initiation to elongation [58]. Similarly, NSD2 is a key contributor to H3K36me2 generation and has also been reported to catalyze H4K20 methylation in certain contexts [51,52,69]. Notably, NSD2 has been shown to increase global levels of H3K36me2 while reducing H3K27me3, leading to large-scale reprogramming of chromatin architecture and transcriptional output, particularly in malignancies such as multiple myeloma [32]. This phenomenon is consistent with the previous observations demonstrating the antagonism between H3K27 and H3K36 methylation [70,71]. Meanwhile, the epigenetic functions of NSD3 remain less well-characterized compared to NSD1 and NSD2. Nonetheless, NSD3 has recently emerged as a key regulator of oncogenic transcriptional program through its robust H3K36me2 activity [22,23].

Beyond their role in active gene transcription, NSD family members are also implicated in transcriptional repression, although the underlying mechanisms are not yet fully understood. In multiple myeloma, NSD2 has been identified as a component of transcriptional corepressor complexes comprising HDACs, mSin3a, and LSD1 demethylase [72]. NSD1 has been reported as a bifunctional transcriptional regulator that may act either as a corepressor or coactivator [73]. More recently, knockout of *Nsd1* in mouse embryonic stem cells was shown to reduce H3K36me2 and impair HDAC1 recruitment, leading to increased H3K27 acetylation at active enhancers and upregulation of target genes [74]. These findings suggest that NSD family may function either as transcriptional activators or repressors in a context-dependent manner to safeguard gene expression programs.

Recent evidence has demonstrated that H3K36 methylation catalyzed by the NSD family serves as a key chromatin signal to guide de novo DNA methylation. Genome-wide analysis revealed that NSD1-mediated H3K36me2 recruits DNMT3A to deposit DNA methylation in intergenic euchromatin regions, a process dependent on the PWWP domain of DNMT3A for recognition of H3K36me2 [75]. Another study further confirmed that the PWWP domain of DNMT3A preferentially binds to H3K36me2 to establish the DNA methylation landscape [76]. These findings suggest that NSD family plays a crucial role in maintaining proper DNA methylation patterns across the genome.

Collectively, these findings highlight NSD family methyltransferases as central epigenetic regulators that coordinate histone and DNA methylation landscapes to control transcriptional output.

#### 2.3.2. Regulation of Development and Cell Differentiation

The NSD family plays pivotal roles in developmental process. Both NSD1 and NSD2 are required for normal embryogenesis, as homozygous loss of either gene in mice leads to lethality with severe developmental defects [68,77]. *Nsd1* deficiency causes early embryonic death around day E10.5 due to mesodermal abnormalities [68], whereas *Nsd2*-null mice survive longer but die shortly after birth with respiratory defects [77]. Beyond embryogenesis, *Nsd1* contributes to germline regulation, with male-specific deficiency leading to spermatogenic failure [78], while *Nsd2* is essential for hematopoietic lineage specification, particularly B cell development, as revealed by stem cell–specific knockout models [79]. In humans, *NSD1* haploinsufficiency underlies Sotos syndrome [80], a congenital overgrowth disorder, and its deletion or point mutation has also been implicated in Beckwith–Wiedemann syndrome [81]. Meanwhile, heterozygous deletion of *NSD2* causes Wolf–Hirschhorn syndrome, displaying delayed growth and development [37]. Although NSD3 is less well characterized in developmental contexts, several evidence highlights its importance in neural crest formation [82,83,84], supporting its potential roles in vertebrate development.

#### 2.3.3. Regulation of Genomic Stability and DNA Damage Response

It has been reported that H3K36 methylation is critical for the maintenance of genomic stability by regulating DNA damage response (DDR) pathway. Upon induction of DNA double-strand breaks (DSBs) by ionizing radiation, H3K36me2 rapidly increases at the damage sites, facilitating the recruitment of early DNA repair components and promoting efficient non-homologous end-joining (NHEJ) repair [85]. Consistently, NSD2-mediated H3K36me2 promotes classical NHEJ at dysfunctional telomeres, thereby protecting genome integrity [86]. In addition to its canonical histone targets, NSD2 can methylate PTEN protein at K349 that is recognized by 53BP1 for PTEN recruitment to DNA damage sites and further efficient repair of DNA DSBs [87]. NSD2 also regulates H4K20 methylation and the accumulation of 53BP1 at DNA DSB sites, further reinforcing its function in the DNA damage response [88]. NSD3, another member of the family, contributes to DNA repair by maintaining H3K36 methylation, which supports homologous recombination (HR) and PARP inhibitor resistance [89]. More recently, isoform-specific function of NSD3 in maintaining genomic stability has been identified. During replication stress, NSD3S localizes to stalled forks and prevents degradation of nascent DNA by MRE11, thereby ensuring fork integrity [90]. Collectively, these findings highlight the essential role of NSD family as chromatin modifiers and scaffolding factors for DNA repair machinery, establishing a mechanistic bridge between H3K36me2 deposition, chromatin homeostasis, and DNA repair fidelity across diverse cellular contexts.

## 3. Genetic Alterations of NSD Family Genes in Human Cancers

**Figure 2:**
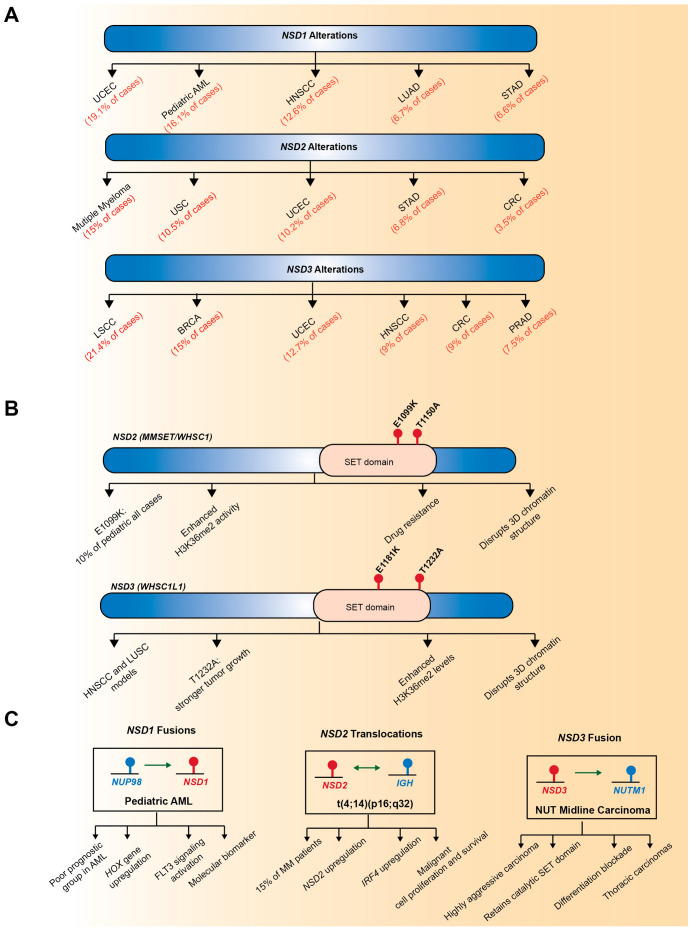
Genetic alterations of NSD family members in human cancer. (**A**) Representative genetic alterations in NSD1, NSD2, and NSD3 across diverse cancer types. NSD1 is frequently involved in chromosomal translocation, forming the NUP98–NSD1 fusion in pediatric acute myeloid leukemia (AML). NSD2 is overexpressed through the t(4;14)(p16;q32) translocation in multiple myeloma. NSD3 is recurrently amplified at the 8p11.23 chromosomal region, leading to overexpression in multiple solid tumors, including breast invasive carcinoma and lung squamous cell carcinoma. (**B**) Point mutations in SET domains. *NSD2* harbors activating mutations including E1099K and T1150A, both enhancing H3K36me2 activity. *NSD3* contains analogous mutations E1181K and T1232A in head and neck squamous cell carcinoma (HNSCC) and lung squamous cell carcinoma (LUSC) models, with T1232A promoting stronger tumor growth and enhanced H3K36me2 levels. (**C**) Chromosomal translocations and fusion events. NUP98-NSD1 fusion occurs in pediatric AML, creating a poor prognostic group characterized by *HOX* gene upregulation and FLT3 signaling activation. The t(4;14)(p16;q32) translocation in multiple myeloma results in NSD2-IGH fusion, leading to NSD2 upregulation, IRF4 upregulation, and enhanced malignant cell proliferation and survival. NSD3-NUTM1 fusion characterizes NUT midline carcinoma, a highly aggressive tumor that retains the catalytic SET domain, blocks differentiation, and commonly presents as thoracic carcinomas. Red text indicates specific genetic alterations, mutations, and associated diseases; Blue bars/boxes represent genes and functional protein domains; Beige/tan ovals highlight the SET domain region. Arrow meanings: Unidirectional arrows (→) indicate causative relationships or functional consequences from genetic alteration to disease/phenotype; Bidirectional arrows (↔) represent chromosomal translocations creating reciprocal gene fusions, where both genes contribute to the fusion protein.

Members of the NSD family have emerged as key oncogenic drivers, showing recurrent alterations such as gene amplifications, point mutations, and chromosomal translocations across diverse human cancers (Figure 2). NSD3 is predominantly dysregulated through gene amplification, whereas NSD1 and NSD2 are more frequently altered by point mutations or translocations. These genetic alterations affect both their catalytic activity towards H3K36 and their non-catalytic scaffold functions, thus exerting widespread influence on cancer development and progression [7,8,9].

### 3.1. Gene Amplification

Among the NSD family members, NSD3 is most prominently dysregulated through gene amplification, leading to its overexpression in a variety of human cancers [7]. In contrast, NSD1 and NSD2 are less often amplified and are instead more commonly affected by point mutations, deletions, or structural variants, suggesting distinct mechanisms of dysregulation within the NSD family (Figure 2A). Notably, *NSD3* is located on chromosome 8p11.23, a genomic region recurrently amplified in several solid tumors, including breast cancer and lung squamous cell carcinoma [25,91,92,93]. Clinically, tumors harboring 8p11.23 amplification exhibit poor prognosis and distinct molecular features, highlighting the potential of *NSD3* gene amplification as a predictive biomarker [91,92,93]. Comprehensive analysis of The Cancer Genome Atlas (TCGA) datasets has identified *NSD3* as a putative druggable cancer driver gene, showing recurrent amplification across multiple cancer types. Specifically, *NSD3* amplification occurs in approximately 21% of lung squamous cell carcinomas, 15% of breast invasive carcinomas, and 5% of ovarian serous cystadenocarcinoma cases [24]. Within this amplicon, NSD3 has emerged as a primary driver oncogene [22,23,94], suggesting that *NSD3* amplification is not a mere bystander event but instead drives functional alterations that promote tumor initiation and progression.

### 3.2. Point Mutations

Recent integrative genomic analyses have revealed that somatic point mutations in NSD family members constitute a distinct and functionally significant class of genetic alterations that reprogram the epigenetic landscape of cancer cells (Figure 2B). These mutations, which frequently occur within the conserved catalytic SET domain, typically act as gain-of-function lesions that enhance the methyltransferase activity of NSD proteins and drive oncogenic transcriptional reprogramming [19,20,23,95,96].

A representative mutation, E1099K, located within the catalytic SET domain of NSD2, has been identified as a gain-of-function mutation that aberrantly enhances NSD2-mediated H3K36me2 activity. This mutation was first discovered in pediatric ALL, where it induced global increases in H3K36me2 levels and reshaped transcriptional programs of leukemia cells [19,20]. Another functionally relevant mutation, NSD2-T1150A, also located within the SET domain, has been identified in mantle cell lymphoma [97]. Structural analysis revealed that both E1099K and T1150A disrupt the autoinhibitory loop that maintains NSD2 in an inactive state, thereby enhancing its catalytic activity toward H3K36 [96]. More recently, NSD2-T1150A was shown to enable H3K36me3, beyond its canonical dimethylation activity [98]. In contrast to these gain-of-function mutations, loss-of-function alterations of *NSD2* have also been reported. In microsatellite instability-high (MSI-H) colorectal cancers, NSD2 undergo frameshift mutations, resulting in the loss of protein expression [99].

Although NSD3 is primarily dysregulated by gene amplification, it also harbors activating mutations within its catalytic SET domain reported in several solid tumors, including head and neck squamous cell carcinoma (HNSCC), lung squamous cell carcinoma (LUSC), and pancreatic cancer cells [23,57,100]. The NSD3 T1232A mutation identified in LUSC is a hyperactive catalytic variant that enhances H3K36me2 levels by increasing the mobility of an autoregulatory loop and improving substrate accessibility, thereby accelerating tumorigenesis in vivo [23]. NSD3 E1181K has also been reported as a gain-of-function alteration within the SET domain, enhancing H3K36 methylation activity [57]. Although single E1181K or T1232A mutations individually increase catalytic activity, their combined occurrence produces a synergistic hyperactive phenotype with markedly enhanced tumorigenic capacity and the highest catalytic activity in HNSCC models.

Together, these findings highlight gain-of-function mutations in NSD family genes as critical epigenetic drivers of tumorigenesis, positioning NSD2 and NSD3 as promising biomarkers and therapeutic targets for cancer treatment.

### 3.3. Chromosomal Translocation

Beyond gene amplification and point mutations, chromosomal translocations affecting NSD family members provide another important mechanism contributing to cancer pathogenesis (Figure 2C). NSD1 is the most well-characterized member with respect to translocation-driven structural variants. In pediatric AML, the *NSD1-NUP98* fusion gene, resulting from a t(5;11)(q35;p15.5) translocation, was first identified [14]. This rearrangement fuses the C-terminal portion of NSD1, which retains the catalytic SET domain, to the FG-repeat domain of nucleoporin-98 (NUP98), a nucleoporin protein family member that mediates interactions with histone acetyltransferases. The NSD1-NUP98 fusion is associated with poor prognosis and defines a distinct AML subgroup with unique clinical and molecular features [15,16]. While translocations involving NUP98 were initially believed to occur with relatively low frequency, re-evaluation of cytogenetically normal (CN)-AML cases revealed that NUP98-NSD1 fusion represents approximately 16% and 2.3% of pediatric and adult cases, indicating frequent misclassification by routine karyotyping and a higher incidence in pediatric AML [15]. NUP98-NSD1 is an independent prognostic factor, and the NUP98-NSD1-positive AML shows distinct *HOX* gene expression patterns compared to MLL-rearranged AML.

The t(4;14)(p16;q32) translocation, found in more than 20% of multiple myeloma cases, generates a novel IGH-NSD2 hybrid transcript, resulting in marked upregulation of NSD2 expression and activity [61,101]. NSD3 also undergoes chromosomal translocations, identified in both hematologic and solid malignancies. In AML patients, the t(8;11)(p11.2;p15) translocation produces an NSD3-NUP98 fusion [102], although its functional consequences remain to be elucidated. Notably, in NUT midline carcinoma, a highly aggressive squamous cell carcinoma characterized by rearrangements of the *NUT/NUTM1* gene, a novel NSD3-NUT fusion oncogene has been identified. This fusion retains the catalytic SET domain of NSD3 and promotes oncogenic transcriptional activation and global chromatin remodeling, leading to a blockade of differentiation and uncontrolled proliferation [50,103].

Collectively, these genetic alterations, including gene amplification, point mutations, and chromosomal translocations, represent functionally significant driver events in the NSD family, emphasizing their contribution to tumorigenesis rather than passive, incidental mutations.

## 4. Diverse Role of NSD Family Methyltransferases in Human Cancer

**Figure 3:**
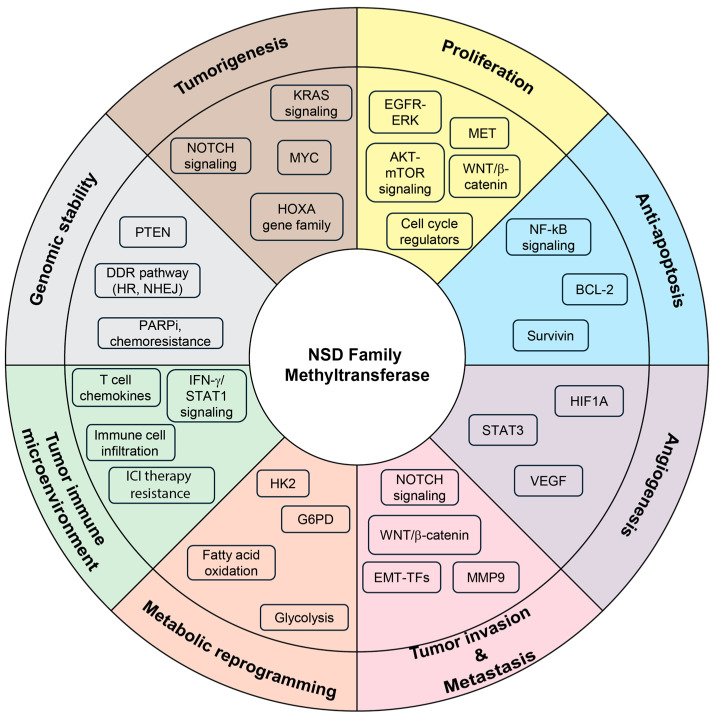
Diverse oncogenic functions of NSD family methyltransferase across cancer hallmarks. Central diagram summarizing the multifaceted roles of NSD family methyltransferases (NSD1, NSD2, and NSD3) in cancer biology. Altered histone modifications (H3K36me1/2, H3K27me3, and H3 acetylation), chromatin remodeling, and protein modifications by NSD family members reprograms transcriptional networks to promote multiple cancer-associated processes, including tumorigenesis (*HOXA* gene family, MYC, KRAS signaling, NOTCH signaling), enhanced cell proliferation (EGFR–ERK, AKT–mTOR, MET, WNT/β-catenin signaling, and cell cycle regulators), resistance to apoptosis (NF-κB signaling, BCL2, and Survivin), angiogenesis (HIF1A, STAT3, VEGF), tumor invasion and metastasis (NOTCH signaling, WNT/β-catenin, EMT-TFs, MMP9), metabolic reprogramming (glycolysis, fatty acid oxidation, HK2, G6PD), modulation of the tumor immune microenvironment (T-cell chemokines, IFN-γ/STAT1 signaling, immune cell infiltration, immune checkpoint inhibitor resistance), genomic stability (PTEN, DDR pathways including HR and NHEJ, PARP inhibitor chemoresistance). Collectively, these pathways highlight the central role of NSD family methyltransferases in coordinating key cancer hallmarks, suggesting them as promising therapeutic targets.

Increasing evidence demonstrates that the NSD family of histone methyltransferases plays a pivotal role in tumor initiation and progression by orchestrating transcriptional activation, chromatin remodeling, and epigenetic reprogramming. Through these mechanisms, NSD proteins contribute to fundamental hallmarks of cancer, including uncontrolled proliferation, survival, invasion, metastasis, maintenance of genomic stability, and immune evasion. Furthermore, they reshape the tumor microenvironment and influence therapeutic responsiveness, underscoring their multifaceted roles in modulating human cancers (Figure 3 and Table 1).

### 4.1. Tumorigenesis, Cancer Cell Proliferation, and Anti-Apoptosis

#### 4.1.1. Roles of NSD1

Members of the NSD family are pivotal regulators of early tumor progression, driving malignant transformation, tumor growth, and survival through the modulation of H3K36 methylation, as well as other various epigenetic and post-translational mechanisms (Table 1).

NSD1, which is recurrently dysregulated by chromosomal translocation in hematopoietic malignancies, is closely linked to leukemogenesis in AML. In this context, the NUP98-NSD1 fusion, characterized by enhanced catalytic activity toward H3K36, drives leukemogenesis by preventing EZH2-mediated repression of *HOXA* genes [17]. The NUP98-NSD1 oncoprotein also interacts with multiple histone-modifying or nucleosome remodeling factor complexes to activate *HOX* gene transcription during leukemogenesis [104,105]. In NUP98-NSD1-positive AML, the fusion protein associates with SMARCA5, a member of the ISWI family of ATP-dependent chromatin remodeler, and its co-dependent partners BPTF and NUP188; catalytically active SMARCA5 is required for NUP98-NSD1-mediated hematopoietic transformation [104]. In addition, NUP98-NSD1 physically interacts with mixed lineage leukemia 1 (MLL1) and non-specific lethal (NSL) histone-modifying complexes to transactivate *HOXA/HOXB* cluster genes, further promoting leukemogenesis [105].

In several solid tumors, tumor-promoting roles of NSD1 have also been reported. NSD1 is overexpressed in hepatocellular carcinoma (HCC) and breast tumor tissues and correlates with poor prognosis [106,107]. NSD1 knockdown suppresses cell proliferation by inhibiting the WNT/β-catenin signaling pathway, accompanied by reduced global H3K36me2 levels and a concomitant increase in H3K27me3 at the promoter region of *WNT10B*. Xenograft models further support the tumor-promoting function of NSD1 in both HCC and breast cancer. In colorectal cancer, lysine methylation of the NF-kB subunit p65 at K218 and K221 is reversibly regulated by NSD1 and the lysine demethylase FBXL11, activating NF-kB signaling and promoting cancer cell growth and colony-forming capacity [108].

In contrast, additional evidence supports tumor-suppressive functions of NSD1. NSD1 inactivation inhibits GATA1-mediated erythroid differentiation, leading to erythroleukemia in mice [109]. These effects depend on the catalytic activity of NSD1, as enforced expression of NSD1 wild type, but not its catalytically inactive mutant (NSD1-N1918Q), induces terminal erythroid maturation with recovered expression of GATA1-target genes in *Nsd1*^−/−^ mice. Furthermore, in a subset of human papillomavirus (HPV)-negative HNSCCs, impaired H3K36 methylation caused by either histone H3K36M mutations or NSD1 loss-of-function alterations blocks cellular differentiation and enhances tumorigenicity [110].

These seemingly contradictory roles of NSD1 can be explained by several molecular determinants. While NSD1 functions as an oncogenic driver when fused to NUP98, its loss-of-function mutations disrupt lineage-specific transcriptional programs such as GATA1-mediated differentiation, thereby accelerating tumorigenesis. These bidirectional roles of NSD1 may be influenced by mutation types, co-factor interactions, and chromatin states in a cancer-type-specific manner.

#### 4.1.2. Roles of NSD2

In multiple myeloma tumors with the recurrent t(4;14) translocation, NSD2 overexpression drives oncogenic transformation of primary cells through the H3K36me2-dependent transcriptional programming [41]. In t(4;14) multiple myeloma cells, NSD2 induces a global increase in H3K36me2 and a concomitant decrease in H3K27me3, establishing an open chromatin state that activates genes involved in cell cycle regulation and the p53 signaling pathway [52].

The gain-of-function mutation of NSD2 E1099K in pediatric ALL significantly contributes to tumorigenesis. In non-transformed ALL cell lines, ectopic expression of this variant representing increased catalytic activity promotes malignant transformation and cell proliferation [20]. Another study also supports that the activating NSD2 mutation aberrantly activates transcription programs involved in specific lineages and adhesion genes, thus increasing proliferation, clonogenicity, and migration in ALL [95]. Likewise, in osteosarcoma, the gain-of-function NSD2 mutations within the SET domain, including E1099K and Y1179A, exhibit a catalytically hyperactive state and promote tumor growth both in vitro and in vivo [57].

NSD2 is also frequently upregulated in diverse solid tumors, including stomach, colon, small cell lung cancers, compared with normal tissues, supporting its role as a putative oncoprotein [111]. In several cancer cell lines with elevated NSD2 expression, NSD2 knockdown inhibits the WNT/β-catenin/TCF-4 pathway and its downstream cyclin D1 expression through reduced H3K36me3, leading to impaired cell cycle progression [112]. NSD2 overexpression also increased the expression of cyclin D, BCL2, and survivin via the positive feedback loop between NSD2 and NF-kB signaling, and promoted tumor growth and survival in castration-resistant prostate cancer [113]. A recent study also demonstrated that NSD2 plays a pivotal role in prostate tumorigenesis, where NSD2-mediated H3K36me2 is required for androgen receptor (AR) transactivation through de novo formation of tumor-specific AR enhancers in AR-dependent prostate cancer [114]. In colorectal cancer, NSD2 overexpression accelerates cell proliferation and migration by increasing global H3K36me2 levels and several cancer-promoting genes, such as *ADAM9*, *EGFR*, and *MET* [115]. NSD2 also exerts oncogenic functions in cervical cancer by promoting cell proliferation, migration, and invasion through activation of the AKT-MMP2 signaling pathway [116].

Interestingly, NSD2 exhibits opposing roles in KRAS-driven tumors. In lung cancer cells, inhibition of NSD2-mediated H3K36me2 results in growth retardation by suppressing KRAS-driven transcriptional program [117]. Similarly, the gain-of-function NSD2 E1099K with elevated H3K36me2 levels activates gene expression program associated with oncogenic KRAS signaling, thus promoting malignant tumor progression in lung adenocarcinoma (LUAD) [118]. Conversely, NSD2 suppresses KRAS-driven pancreatic tumorigenesis by inducing the H3K36me2-dependent expression of IκBα, an inhibitor of NF-κB signaling, suggesting a putative tumor suppressive role [119]. These findings suggest a context-dependent function of NSD2 in KRAS-driven tumors.

Overall, despite some controversies in KRAS-driven tumors, NSD2 appears to function as a critical epigenetic driver that promotes tumorigenesis across both hematologic and solid malignancies.

#### 4.1.3. Roles of NSD3

NSD3 was initially identified as a gene capable of driving malignant transformation in breast cancer. Transgenic mouse models overexpressing NSD3 developed mammary gland hyperplasia, dysplasia, and ultimately invasive ductal carcinoma [120]. Screening for transforming properties of genes within the 8p11-12 amplicon in breast cancer also suggested that NSD3 functions as a potential oncogene, although the mechanism involving H3K36 methylation remained unclear [94]. More recently, our group demonstrated that the long isoform of NSD3 (NSD3L), which retains its catalytic SET domain, is essential for promoting breast tumorigenesis. In a xenograft mouse model with MCF10A immortalized mammary epithelial cells, NSD3L exhibited a markedly higher transforming ability compared with the short isoform (NSDS) lacking the SET domain. Mechanistically, NSD3L facilitated H3K36 methylation at the promoter regions of genes involved in the NOTCH signaling pathway, thereby enhancing transcriptional activation that led to NOTCH receptor cleavage and subsequent signaling activation [22]. This study provided the first direct evidence that the methyltransferase activity of NSD3 drives tumor initiation and progression.

A recent study further highlighted the key oncogenic role of NSD3 methyltransferase in LUSC, showing that NSD3 is a stronger cancer driver than FGFR1 within the 8p11-12 amplicon [23]. Elevated NSD3 levels or the NSD3 T1232A gain-of-function mutation increase H3K36me2 deposition, which in turn promotes the transcriptional activation of oncogenic drivers such as *MYC*, thereby facilitating tumorigenesis. Likewise, in HNSCC, the gain-of-function NSD3 mutations that enhance H3K36 methylation increased tumor growth in xenograft models [57], and the impaired function of NSD3 by H3K36 mutation reduced proliferation of HNSCC cells [89]. NSD3 also drives cell cycle progression via the H3K36me2-dependent upregulation of *CDC6* and *CDK2* in HNSCC [121]. Moreover, NSD3 can directly monomethylate EGFR at K721 in the tyrosine kinase domain to activate EGFR-ERK signaling and promote cell cycle progression in this cancer [46]. In NUT midline carcinoma, the NSD3-NUT fusion interacts with BRD4 for BRD4-dependent transcription that is required for blockade of differentiation and sustaining cell proliferation [50]. The tumor-promoting function of NSD3 has also been reported in pancreatic cancer, where NSD3 expression is elevated in tumor tissues. NSD3 knockdown induces cell cycle arrest and apoptosis, while the NSD3 T1232A mutant accelerates cell proliferation and invasion. Mechanistically, NSD3-mediated H3K36me2 upregulates several oncogenic targets, including *MYC*, *ADAM12*, *NOTCH3*, and activates mTOR signaling pathway [100]. Another study revealed that NSD3 depletion in pancreatic cancer cells downregulates EGFR/ERK signaling-associated genes, consistent with reduced H3K36me2 levels [122].

Collectively, increasing evidence establishes NSD3 as a potent oncogenic driver that facilitates tumorigenesis across multiple solid cancers, highlighting its potential as a promising therapeutic target.

### 4.2. Tumor Angiogenesis, Invasion and Metastasis

During cancer progression following tumor establishment, members of the NSD family play key roles in promoting tumor angiogenesis, migration and invasion for metastatic dissemination. Several studies have suggested that NSD proteins may contribute to tumor angiogenesis through their catalytic activity toward both histone- and non-histone substrates. In castration-resistant prostate cancer cells, NSD2-mediated H3K36me2 upregulates the expression of vascular endothelial growth factor-A (VEGF-A), a key regulator of angiogenesis [113]. NSD2 also induces angiogenesis in a catalytic activity-dependent manner by methylating STAT3 protein at K163, thereby enhancing STAT3 activation [44]. In esophageal cancer, NSD1 upregulates HIF-1α expression in collaboration with STAT3, which in turn promotes HIF-1α–driven VEGF signaling and subsequent angiogenic processes [123].

The NSD family further promotes invasion and metastasis by regulating the expression of EMT-related and cell motility-associated genes. In paclitaxel-resistant MCF7 breast cancer cells, NSD1 knockdown inhibits EMT, migration and invasion, while restoring sensitivity to paclitaxel [124]. NSD1 also promotes the migration and invasion of HCC cells by activating WNT signaling through the H3K36me2-dependent transcriptional program [106]. While no direct evidence for regulation of tumor metastasis by NSD1, altered NSD1 expression has been associated with occult lymph node metastasis in patients with HNSCC [125].

NSD2-induced H3K36me2 has been shown to promote EMT by upregulating TWIST1 in both prostate cancer and t(4;14)-positive multiple myeloma [126,127]. In prostate cancer, AKT signaling stabilizes NSD2 protein to enhance its H3K36me2 activity and subsequent transcriptional programs that support invasive behavior. This cooperation increases metastatic potential, as demonstrated in PTEN-deficient mouse models with elevated NSD2 expression [128]. Another study also identified that NSD2 is a critical cancer driver facilitating metastasis of prostate cancer [129]. NSD2 is also closely linked to metastatic progression in human breast cancer. In breast cancer cells, NSD2 activates WNT/β-catenin signaling to facilitate EMT and invasion [130]. Furthermore, in MDA-MB-231 cells and clinical samples, TGF-β and TNF-α synergistically induce MMP9 expression via NSD2-mediated H3K36me2, promoting invasion and metastasis [131]. A recent report further supports the pivotal role of NSD2-H3K36me2 axis in promoting metastasis of triple-negative breast cancer (TNBC) [132]. Consistent with these findings, NSD2 knockdown inhibits EMT, migration and invasion, accompanied by increased E-cadherin in renal cell carcinoma and osteosarcoma [133,134].

Our previous work identified NSD3 as a driver of metastatic progression in human breast cancer. Clinically, high NSD3 expression correlates with tumor recurrence by distant metastasis. In MCF7 cells, NSD3 overexpression induces EMT and invasion, while NSD3 knockdown in MDA-MB-231 cells reverses EMT, suppresses migration and invasion, and reduces tumor metastasis in vivo. Mechanistically, NSD3 enhances H3K36 methylation at the promoters of EMT and NOTCH pathway–related genes, thereby upregulating their transcription [22].

Together, these findings highlight the crucial roles of NSD family members in driving the acquisition of aggressive phenotypes beyond initial tumor formation across diverse human cancers.

### 4.3. Metabolic Reprogramming

Emerging evidence indicates that NSD family members contribute to the metabolic rewiring of human cancers. A recent study identifying metabolism-related subtype of laryngeal cancer (LCA) indicated that the LCA1 subtype, which exhibits favorable prognosis and enriched metabolic pathway, possesses damaging mutations in the *NSD1* gene [135], while its functional consequences remain to be clarified. In HNSCC, NSD1 mutation was shown to attenuate mitochondrial respiration while enhancing glycolysis via the modulation of TGFB2/PPARGC1A signaling pathway [136].

NSD2-induced H3K36me2 also upregulates key enzymes in glucose metabolism, including hexokinase 2 (HK2) and glucose-6-phosphoate dehydrogenase (G6PD), in endocrine therapy-resistant cancer cells [137]. In t(4;14) multiple myeloma, NSD2 overexpression promotes PKCα-driven metabolic processes that increases HK2 expression [138], or disrupts the synthesis of creatine by diverting SAM to global H3K36me2, thereby creating higher dependence on adenylate kinase 2 (AK2), a mitochondrial enzyme catalyzing the transfer of high-energy phosphate [139]. Moreover, NSD2 directly methylates active regulator of SIRT1 (AROS) at K27 to facilitate its interaction with SIRT1, resulting in SIRT1 activation and enhanced fatty acid oxidation in colorectal cancer [45].

Contrary to the finding in LUSC where NSD3 functions as an oncogenic driver, NSD3 has recently been shown to inhibit proliferation, migration, and invasion in LUAD through metabolic alteration [140]. As a mechanism underlying this event, inhibitory effect of NSD3 on glycolysis by forming a trimer with PPP1CB and p-STAT3 for dephosphorylation of STAT3 and subsequent suppression of HK2 transcription is identified, providing a novel role of NSD3 in modulating metabolic reprogramming. Similarly, the PWWP domain of NSD3S inhibits oxygen consumption and increases lipid peroxidation, driving a metabolic shift from oxidative phosphorylation toward aerobic glycolysis [141].

Although further studies are required to fully understand the role of NSD family in cancer metabolism, current evidence supports the crucial role of NSD family members in modulating glucose and fatty acid metabolisms to sustain tumor growth and survival.

### 4.4. Tumor Immune Microenvironment

Although still limited, growing evidence demonstrates the potential role of NSD family members in modulating the tumor immune microenvironment [142]. In squamous cell carcinomas, loss or inactivation of NSD1 occurs frequently and is associated with reduced immune infiltration and impaired interferon responses [143]. Likewise, NSD1-deficient HNSCCs with reduced H3K36me2 exhibit low levels of T cell chemokines, decreased T cell infiltration, and poor response to anti-PD-1 therapy, whereas inhibition of KDM2A, a demethylase for H3K36me, can reverse these effects [144]. Multi-omics analyses also identified NSD1 as one of the genes positively correlated with tumor mutation burden (TMB), a predictive biomarker for immunotherapy response [145]. These results suggest that NSD1 may contribute to inflamed tumor microenvironment and thereby enhance responsiveness to immune checkpoint inhibitors.

Among NSD family members, NSD2 has been more widely implicated as a regulator of immune cells in the tumor microenvironment. In prostate cancer, high NSD2 expression is associated with immunosuppressive phenotype, whereas NSD2 inhibition increases tumor-infiltrating CD8+ T cells [146]. Another study reported that NSD2 expression correlated positively with CD4+ T cell infiltration but negatively with CD8+ T cell infiltration [147]. In colorectal cancer, NSD2 silencing downregulates MHC-1 levels and inactivates IFN-γ/STAT1 signaling, thereby impairing antitumor immunity [148]. In hepatocellular carcinoma, NSD2 expression correlates with immune cell infiltration, including B cells, CD4+ T cells, Tregs, and macrophages [149]. In KRAS-driven lung cancer, inactivation of NSD2 by the oncohistone H3K36M leads to activation of an antiviral-like immune response through SETD2-mediated H3K36me3 deposition [150].

While the role of NSD3 in tumor immunity remains largely unexplored, several clinical studies suggest its potential importance in reprogramming the tumor immune microenvironment. In patients with breast cancer, high NSD3 expression was associated with elevated PD-L1 levels and reduced CD8+ T cell infiltration [151]. NSD3-amplified LUSC exhibits a non-inflamed tumor immune microenvironment with a worse immunotherapy outcome [152]. In contrast, in pancreatic cancer, NSD3 expression correlates positively with immune cell infiltration [122]. A previous study reported that NSD3 directly methylates interferon regulatory factor 3 (IRF3) at K366 for enhancing IRF3-mediated anti-viral innate immunity [42]. Given that IRF3 is a key component for recognition of tumor antigen during the innate immunity, this finding raises the possibility that NSD3 may play a critical role in tumor immune surveillance.

### 4.5. Regulation of Genomic Stability and Response to Chemotherapy

H3K36 methylation by NSD family members is a critical event that preserves genome stability under normal physiological conditions. In human cancers, they also play a key role in maintaining genomic integrity in response to DNA damage.

In HNSCCs, a subset of tumors harboring reduced H3K36 methylation by H3K36 mutations with concomitant elevation of H3K27me3 levels exhibit higher genomic instability and increased sensitivity to PARP1/2 inhibitors. These effects are attributed to impaired BRCA1- and FANCD2-dependent DNA repair pathways, supporting the notion that the balance between H3K36 and H3K27 methylation is essential for maintaining genomic stability [89]. In this cancer model, H3K36M interacts with NSD2 and NSD3, thus inhibiting their enzymatic activities toward H3K36 methylation. Another study showed that NSD1 disruption in HPV-negative HNSCC enhances sensitivity to cisplatin, a DNA-damaging platinum-based chemotherapy agent, by global DNA hypomethylation [153]. Consistently, patients harboring NSD1 inactive mutation displayed improved survival in HNSCC [153].

NSD2 overexpression with elevated H3K36me2 levels is closely associated with enhanced DNA damage repair and resistance to cytotoxic chemotherapy. In U2OS cell line system, NSD2 activates both NHEJ and HR repair pathways, while loss of NSD2 decreases the expression and recruitment of DNA repair proteins at sites of DNA DSBs [154]. Moreover, in t(4;14) multiple myeloma, high NSD2 expression confers resistance to DNA-damaging agents by maintaining an intact DNA repair system, while NSD2 inhibition enhances chemosensitivity [154]. NSD2 also dimethylates PTEN at K349, which facilitates 53BP1 recruitment to sites of DNA damage and promotes the resolution of DSBs through γH2AX dephosphorylation. The inhibition of NSD2-mediated PTEN methylation sensitizes tumor cells to DNA-damaging agents in combination with a PI3K inhibitor in colorectal cancer. This positions NSD2 as a critical integrator of the DNA damage response and PI3K signaling, providing a rationale for combination therapy strategies using PI3K inhibitors and DNA-damaging agents in tumors with elevated NSD2 activity [87].

Collectively, this evidence underscores the NSD family as key modulators of DDR pathways, suggesting that targeting these enzymes may enhance the efficacy of DNA-damaging anti-cancer therapies.

### 4.6. Therapeutic Resistance

In addition to DNA-damaging agents, the expression and activities of NSD family members are linked to therapeutic resistance in diverse types of human cancers.

NSD1 is upregulated in paclitaxel-resistant MCF7 breast cancer cells, and its knockdown in these cells attenuates cell growth and induces apoptosis [107]. NSD2 contributes to endocrine resistance in hormone receptor-positive breast cancer. NSD2-mediated metabolic reprogramming confers resistance to tamoxifen, an anti-estrogen [137], and treatment with DZNep, an indirect inhibitor of methyltransferases, induces NSD2 degradation, thus restoring tamoxifen sensitivity in breast cancer [155]. Alternative splicing of NSD2 mRNA with the inclusion of its exon 2 due to downregulation of nuclear speckle splicing regulatory protein 1 (NSRP1) has also been shown to drive resistance to CDK4/6 inhibitors in hormone receptor-positive breast cancer [156]. In multiple myeloma, metabolic reprogramming by NSD2-PKCα axis induces resistance to lenalidomide, an immunomodulatory agent [138]. In addition, NSD2-induced stabilization of steroid receptor coactivator 3 (SRC3) is involved in resistance to bortezomib, a proteasomal inhibitor used in multiple myeloma therapy, while disrupting the interaction between NSD2 and SRC3 recovers the sensitivity to bortezomib [157]. Meanwhile, in NSD2-high multiple myeloma harboring t(4;14) translocation, all-trans retinoic acid (ATRA) induces the expression of CD38, in a dependent manner of NSD2-mediated H3K36me2, thus improving the efficacy of anti-CD38 CAR T-cell therapy [158]. NSD2 has also been implicated in modulating melanoma responsiveness to romidepsin and interferon-α2b treatment [159].

Overall, although further investigations are needed to fully elucidate NSD family-mediated mechanisms of drug resistance, these findings underscore the therapeutic potential of targeting NSD enzymes to overcome resistance and enhance the efficacy of conventional anti-cancer therapies.

**Table 1:** Key functions and related mechanisms of NSD family members in human cancer.

Malignant Phenotypes	NSD Family Member	Cancer Types	Functional Roles	Molecular Mechanisms		Refs.
				Type of Mechanism	Mode of Action (Target Genes, Proteins, and Signaling)	
Tumorigenesis, Proliferation, and Survival	NSD1	acute myeloid leukemia (NUP98-NSD1 fusion)	tumor-promoting	histone modification, chromatin remodeling	NUP98-NSD1 fusion promotes leukemogenesis by H3K36me2-mediated *HOXA* gene upregulation; inhibits EZH2-mediated H3K27me3; interacts with chromatin remodeler complexes	[17,104,105]
		hepatocellular carcinoma, breast cancer	tumor-promoting	histone modification	upregulates Wnt10b expression by inhibiting H3K27me3 and subsequently activates WNT/β-catenin signaling pathway	[106,107]
		colorectal cancer	tumor-promoting	protein methylation	methylates p65 at K218/221 and activates NF-κB signaling to promote cell proliferation	[108]
		erythroleukemia	tumor-suppressive	histone modification, transcriptional regulation	NSD1 inactivation impairs GATA1-mediated erythroid differentiation, leading to leukemogenesis	[109]
		head and neck squamous cell carcinoma	tumor-suppressive	histone modification	Impaired NSD1 activity by loss-of-function mutation or H3K36M blocks cellular differentiation, thus promoting oncogenesis	[110]
	NSD2	multiple myeloma harboring t(4;14)	tumor-promoting	histone modification	NSD2-induced H3K36me2 drives oncogenic transcriptional program to promote tumorigenesis, proliferation, and anti-apoptosis; inhibits H3K27me3	[41,52]
		acute lymphoblastic leukemia, osteosarcoma	tumor-promoting	histone modification	NSD2 hyperactivation by gain-of-function mutation (E1099K/Y1179A) drives H3K36me2-dependent oncogenic transcriptional program	[20,57,95]
		bladder cancer, lung cancer	tumor-promoting	protein interaction, histone modification	promotes cell proliferation by interacting with WNT pathway components and activating WNT/β-catenin/TCF4 signaling (cyclin D1 upregulation)	[112]
		prostate cancer	tumor-promoting	protein interaction, histone modification	upregulates cyclin D, BCL2, and survivin via NF-κB signaling; activates androgen receptor (AR)-dependent transcription to drive prostate tumorigenesis	[113,114]
		colorectal cancer, cervical cancer	tumor-promoting	histone modification	upregulates cancer-promoting genes (e.g., *ADAM9*, *EGFR*, and *MET*) by H3K36me2; activates PI3K-AKT pathway	[115,116]
		lung adenocarcinoma	tumor-promoting	histone modification	regulates KRAS-driven transcriptional program, activates KRAS-MEK pathway	[117,118]
		pancreatic cancer	tumor-suppressive	histone modification	inhibits tumorigenesis by H3K36me2-mediated IκBα upregulation and subsequent inactivation of NF-κB signaling	[119]
	NSD2-S	t(4;14) multiple myeloma	tumor-promoting	transcriptional regulation	NSD2S alone is sufficient to drive tumorigenesis; upregulates glyoxalase 1 (GLO1) to induce anti-apoptotic genes (MCL2 and BCL2)	[27]
	NSD3	breast cancer	tumor-promoting	histone modification	NSD3-induced H3K36me2 promotes tumorigenesis by upregulating NOTCH pathway and EMT-related genes	[22]
		lung squamous cell carcinoma	tumor-promoting	histone modification	NSD3 amplification or GOF mutation (T1232A) induces H3K36me2-dependent oncogenic transcriptional program (MYC upregulation, mTOR activation)	[23]
		head and neck squamous cell carcinoma	tumor-promoting	histone modification, protein methylation	NSD3 GOF mutation drives oncogenic transcriptional program; upregulates cell cycle regulators (CDC6, CDK2); methylates EGFR at K721 for its activation	[46,89,121]
		NUT midline carcinoma (NSD3-NUT fusion)	tumor-promoting	protein interaction, transcriptional regulation	NSD3-NUT fusion blocks cell differentiation and maintains proliferation by interacting with BRD4 for BRD4-dependent transcription	[50]
		pancreatic cancer	tumor-promoting	histone modification	upregulates MYC, ADAM12, and NOTCH3; activates mTOR, EGFR/ERK signaling	[100,122]
	NSD3-S	acute myeloid leukemia	tumor-promoting	chromatin remodeling	BRD4-NSD3S-CHD8 complex binds to super-enhancer of *MYC* gene for its transcription that is required for sustaining AML	[26]
		breast cancer, lung cancer	tumor-promoting	transcriptional regulation, protein interaction	regulates ER expression and its estrogen-independent activity; interacts with MYC for its transcriptional activation	[160,161]
Tumor Angiogenesis	NSD1	esophageal cancer	pro-angiogenic	histone modification	upregulates HIF-1α and VEGF-A expression by recruiting STAT3 to HIF-1α promoter and facilitating H3K36me2	[123]
	NSD2	prostate cancer	pro-angiogenic	histone modification	induces H3K36me2-mediated VEGF-A expression	[113]
		colorectal cancer	pro-angiogenic	protein methylation	methylates STAT3 at K163 for activation of STAT3/VEGF-A axis	[44]
Tumor Invasion & Metastasis	NSD1	breast cancer	pro-metastatic	protein methylation	promotes EMT, migration, and invasion by inhibiting FBXL11 to promote p65 methylation and subsequent NF-κB signaling activation	[124]
		hepatocellular carcinoma	pro-metastatic	histone modification	promotes migration, invasion, and metastasis by epigenetic upregulation of Wnt10b for WNT/β-catenin signaling activation	[106]
		head and neck squamous cell carcinoma	pro-metastatic	unknown	is associated with occult lymph node metastasis in HNSCC patients	[125]
	NSD2	t(4;14)+ multiple myeloma	pro-metastatic	transcriptional regulation	upregulates TWIST1 transcription to induce EMT and tumor dissemination	[127]
		prostate cancer	pro-metastatic	histone modification, posttranslational modification	drives EMT, invasion, and metastasis; upregulates H3K36me2-mediated TWIST1 expression; NSD2 protein stabilization by AKT upregulates RICTOR and Rac1 expression via H3K36me2 to drive metastasis	[126,128,129]
		breast cancer	pro-metastatic	histone modification, transcriptional regulation	facilitates EMT, migration, and invasion by activating FOXM1-mediated WNT/β-catenin signaling; upregulates MMP9 and ULK1 expression via H3K36me2	[130,131,132]
		renal cell carcinoma, osteosarcoma	pro-metastatic	transcriptional regulation	promotes EMT, migration, and invasion by downregulating E-cadherin	[133,134]
	NSD3	breast cancer	pro-metastatic	histone modification	promotes EMT, invasion, and metastasis by H3K36me2-dependent activation of NOTCH signaling and EMT program	[22]

## 5. Role of NSD Short Isoforms in Regulating Cancer

**Figure 4:**
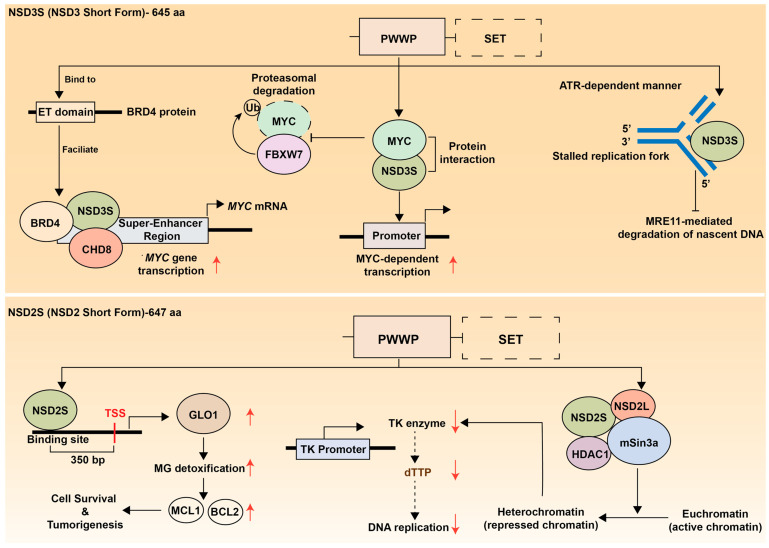
H3K36-independent functions of NSD family short isoforms. (**Upper panel**) NSD3S (NSD3 Short Form, 645 amino acids) mechanisms. The short isoform lacks the catalytic SET domain (shown as dashed box) but retains PHD1 and PWWP domains. NSD3S binds to the ET domain of BRD4 protein, facilitating formation of a BRD4-NSD3S-CHD8 transcriptional complex that promotes MYC mRNA and protein expression. NSD3S also directly interacts with the MYC protein to activate MYC-dependent transcriptional programs. Separately, NSD3S stabilizes MYC by preventing FBXW7-mediated proteasomal degradation. Additionally, NSD3S provides replication fork protection in an ATR-dependent manner by localizing to stalled replication forks and preventing MRE11-mediated degradation of nascent DNA, thereby maintaining genomic stability and contributing to therapy resistance. (**Lower panel**) NSD2S (NSD2 Short Form, 647 amino acids) functions. This isoform also lacks the SET domain but contains PHD1 and PWWP domains. NSD2S acts as both a transcriptional activator and repressor. As a transcriptional activator, LSD2S upregulates GLO1 expression to induce the transcription of anti-apoptotic genes, thereby promoting cancer cell survival in multiple myeloma. Conversely, NSD2S can form a transcriptional repressive complex through interactions with NSD2 long form, HDAC1, and mSin3 proteins. NSD2 binding to the thymidine kinase (TK) promoter reduces TK enzyme expression, which may decrease dTTP production and subsequently impair DNA replication. Red upward and downward arrows indicate the upregulation and downregulation of the adjacent molecules or biological processes, respectively.

While the canonical function of NSD family proteins centers on their methyltransferase activity, emerging evidence reveals that these enzymes exert significant oncogenic effects through mechanisms independent of histone H3K36 methylation. The short isoforms of NSD2 and NSD3, which lack the catalytic SET domain, retain potent tumor-promoting capabilities by functioning as chromatin scaffolds, transcriptional adaptors, and protein interaction hubs (Figure 4 and Table 1). Understanding these H3K36-independent roles is crucial for developing comprehensive therapeutic strategies that address the full spectrum of NSD family contributions to oncogenesis.

### 5.1. NSD3 Short Isoform

The short isoform of NSD3 (NSD3S) lacks the catalytic SET domain and therefore does not possess intrinsic methyltransferase activity. Nevertheless, NSD3S has been identified as an independent transcriptional regulator with potent oncogenic potential.

The crucial oncogenic function of NSD3S was first identified in AML, where the bromodomain and extraterminal (BET) protein BRD4 acts as a key driver of tumorigenesis. In AML, NSD3 is expressed exclusively as a short isoform, and NSD3S plays a pivotal role in maintaining leukemic transcriptional programs. Mechanistically, NSD3S acts as an adaptor protein by directly binding to the ET domain of BRD4, thereby facilitating the formation of a BRD4-NSD3S-CHD8 complex. This complex localizes to super-enhancers and drives the transcriptional activation of critical oncogenes such as *MYC*. Knockdown of either NSD3 or CHD8 reduces *MYC* transcription and induces differentiation of AML cells, indicating that the NSD3-BRD4-CHD8 axis forms a central transcriptional regulatory network sustaining AML cell proliferation and survival [26]. A subsequent study demonstrated that amplification of the NSD3-BRD4-CHD8 pathway also occurs in pelvic high-grade serous carcinoma (HGSC) and is associated with poor prognosis, suggesting this pathway as a potential therpeutic target in a subset of HGSC patients [162].

In a xenograft model using MCF10A immortalized mammary epithelial cells, NSD3S alone has the potential to induce breast tumorigenesis, although its transforming ability is inferior to that of NSD3L [22]. Moreover, in SUM-44 hormone receptor-postive breat cancer cells, overexpression of NSD3S drives estrogen receptor alpha (ERα) activity in an estrogen-independent manner, and its depletion leads to reduced ER expression and impaired cell proliferation [160], highlighting the critical role of NSD3S in breat cancer pathogenesis.

Furthermore, screening for oncogenic protein-protein interactions in lung cancer cells identified a physical interaction between NSD3S and the MYC oncoprotein via its PWWP domain, which enhances MYC transcriptional activity [161]. In addition, another study reported that NSD3S directly binds to and stabilizes MYC protein by preventing FBXW7-mediated proteasomal degradation [163]. Together, these findings suggest that NSD3S functions as a key regulator of MYC transcription, stability, and activity, thereby reinforcing MYC-driven oncogenic transcriptional programs (Figure 4, upper panel).

Beyond transcriptional regulation, NSD3S has also been implicated in maintaining genomic stability. A recent study in prostate cancer demonstrated that NSD3S contributes to replication fork protection during replication stress [90]. Upon ATR activation, NSD3S localizes to stalled replication forks and prevents MRE11-mediated degradation of nascent DNA, thereby preserving fork integrity and limiting DNA damage accumulation (Figure 4, upper right panel). This protective mechanism confers resistance to genotoxic agents such as PARP inhibitors, whereas targeted degradation of NSD3S disrupts fork stability and restores therapeutic sensitivity. Targeted degradation of NSD3S disrupts fork stability and restores sensitivity to therapy, highlighting its critical role in the replication stress response [90].

Taken together, these findings demonstrate the essential role of NSD3S in driving and sustaining tumorigenesis, suggesting that both NSD3L and NSD3S represent attractive therapeutic targets in human cancer.

### 5.2. NSD2 Short Isoform

While the functional role of the SET domain-deficient short isoform of NSD2, known as MMSET I, remains less well characterized than that of NSD3S, accumulating evidence supports its critical contribution to oncogenesis (Figure 4, lower panel). The short isoform of NSD2 (MMSET I) is overexpressed in multiple myeloma harboring the t(4;14) translocation [61]. Although both long and short isoforms are co-expressed in these tumors, MMSET I alone is sufficient to drive cell proliferation and tumor formation [27]. Mechanistically, MMSET I binds to the upstream transcription start site of glyoxalase 1 (GLO1) for its transactivation and increases GLO1-mediated upregulation of anti-apoptotic factors, MCL1 and BCL2.

In t(4;14) multiple myeloma, MMSET I has been implicated in transcriptional repression. MMSET I collaborates with HDAC1 and the mSin3b corepressor complex, leading to suppression of thymidine kinase (TK) gene expression [164]. It also interacts with its long isoform, MMSET II, a component of mSin3A-HDACs-LSD1 corepressor complex [72]. In addition, RE-IIBP, another short isoform of NSD2 that retains catalytic activity, is upregulate in leukemia patients and contributes to transcriptional repression by promoting H3K27 methylation and histone deacetylation [165].

Collectively, these findings underscore the isoform-specific oncogenic functions of NSD family members and suggests that the short variants represent viable therapeutic targets distinct from their catalytically active counterparts.

## 6. Targeting NSD Family for Cancer Treatment

**Figure 5:**
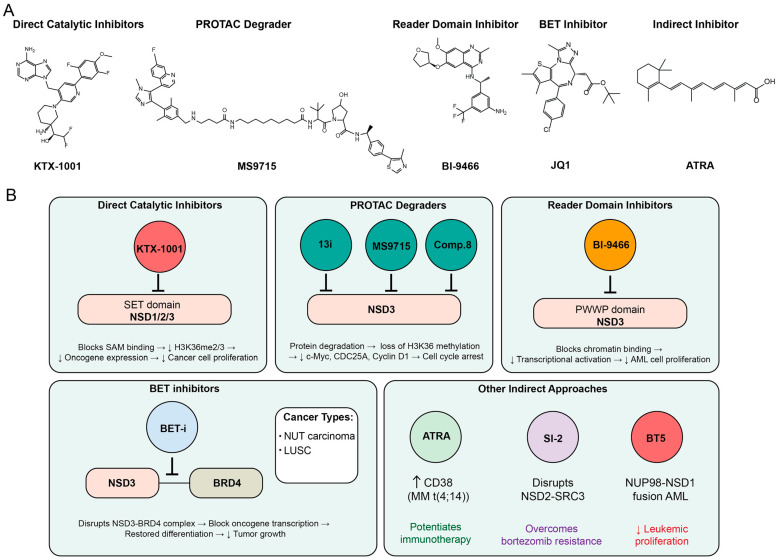
Therapeutic targeting strategies for NSD family proteins in cancer. (**A**) Chemical structures of representative NSD family inhibitors across different therapeutic modalities. KTX-1001 represents direct catalytic SET domain inhibitors that block S-adenosylmethionine (SAM) binding, leading to decreased H3K36me2/3 levels, reduced oncogene expression, and diminished cancer cell proliferation. MS9715 exemplifies PROTAC degraders that induce protein degradation, resulting in loss of H3K36 methylation and decreased expression of key targets including c-Myc, CDC25A, and Cyclin D1, ultimately causing cell cycle arrest. BI-9466 represents reader domain inhibitors that block chromatin binding to the PWWP domain, decreasing transcriptional activation and reducing acute myeloid leukemia (AML) cell proliferation. JQ1 illustrates BET inhibitors that disrupt the NSD3-BRD4 complex, blocking oncogene transcription, restoring differentiation, and reducing tumor growth in NUT carcinoma and lung squamous cell carcinoma (LUSC). ATRA (All-trans retinoic acid) represents indirect approaches that potentiate immunotherapy by enhancing CD38 expression in multiple myeloma with t(4;14) translocation. (**B**) Therapeutic applications and mechanisms of action. Direct catalytic inhibitors (KTX-1001, BT5) target SET domains of NSD1/2/3, with BT5 specifically effective in NUP98-NSD1 fusion AML by reducing leukemic proliferation. PROTAC degraders (13i, MS9715, Compound **8**) provide targeted protein degradation across different cancer types. Reader domain inhibitors (BI-9466) selectively target the NSD3 PWWP domain. BET inhibitors demonstrate efficacy in NUT carcinoma and LUSC by disrupting NSD3-BRD4 interactions. Other indirect approaches include SI-2, which disrupts NSD2-SRC3 interactions to overcome bortezomib resistance in multiple myeloma, and ATRA, which potentiates CD38-targeted immunotherapy in t(4;14) multiple myeloma. Upward and downward arrows indicate up- and down-regulation of adjacent regulators or phenomena, respectively.

The comprehensive understanding of NSD family proteins as central drivers of oncogenesis has catalyzed the development of diverse therapeutic strategies targeting these epigenetic regulators. The multifaceted roles of NSD1, NSD2, and NSD3 in cancer progression ranging from H3K36 methylation-dependent transcriptional activation to methylation-independent scaffolding functions present both opportunities and challenges for therapeutic intervention. Direct catalytic inhibition of the SET domain represents the most straightforward approach, with several small-molecule inhibitors demonstrating preclinical efficacy and advancing into clinical trials. However, the functional complexity of NSD proteins, including their isoform-specific activities and non-histone substrates, has necessitated the exploration of alternative targeting modalities. Protein degradation strategies using PROTAC (PROteolysis-Targeting Chimera) offer the advantage of eliminating both catalytic and non-catalytic functions, while reader domain inhibitors provide selectivity by disrupting specific chromatin interactions. Additionally, indirect approaches targeting NSD-interacting partners or downstream effectors have shown promise in overcoming resistance mechanisms and enhancing the efficacy of existing therapies. As our mechanistic understanding of NSD family biology deepens, the therapeutic landscape continues to evolve, offering multiple avenues for precision medicine approaches tailored to specific genetic contexts and tumor types (Figure 5).

### 6.1. Catalytic Inhibitors for NSD Family

Research on the SET domains of the NSD family members, NSD1, NSD2, and NSD3, has highlighted viable strategies for the rational design of direct catalytic inhibitors in cancer therapy (Table 2). Structural studies of these domains have uncovered distinct regulatory and ligand-binding features that enable the development of subtype-specific inhibitors.

Among the NSD family enzymes, NSD2 has been the most extensively explored for small-molecule inhibitor development, as it represents an attractive therapeutic target in t(4;14)-positive multiple myeloma characterized by aberrant overexpression and hyperactive catalytic function. Initial NSD2-SET inhibitors, such as LEM-06 and LEM-14, exhibited micromolar potencies (IC_50_ = 0.8 mM and 132 μM, respectively) and modest selectivity, serving primarily as chemical tools with limited translational potential [166,167]. A high-throughput screening using nucleosome substrates identified five NSD2-targeting inhibitors—DA-3003-1, PF-03882845, Chaetocin, TC LPA54, and ABT-199—although none showed strong selectivity toward NSD2. Among them, DA-3003-1 demonstrated the most potent inhibitory activity, with an IC_50_ of 0.17 μM [168]. Structural optimization of DA-3003-1 (compound **15a**) further improved anti-proliferative activity in KMS-11 multiple myeloma cells both in vitro and in vivo [169]. A novel NSD2 selective inhibitor, compound **42**, also exhibited pronounced anti-tumor effects in RS4;11 ALL xenograft model with favorable tolerability [170]. These findings suggest that NSD2 catalytic inhibition may become a promising therapeutic strategy for targeting hematologic malignancies.

More recently, several NSD2-selective inhibitors have begun to approach clinical translation. The most advanced NSD2 catalytic inhibitor is KTX-1001, which is currently being evaluated in a Phase I clinical trial for relapsed or refractory multiple myeloma, underscoring the translational potential of this approach [171,172]. Biochemical assays demonstrated that KTX-1001 is highly potent and selective toward NSD2-SET. Notably, KTX-1001 exhibited non-competitive behavior with respect to SAM and nucleosomes, potentially enabling robust potency despite high intracellular SAM concentration. In addition, recently developed clinical-grade NSD2 inhibitors, IACS-17596 and IACS-17817, synthesized based on the patent disclosing KTX-1001, display nanomolar potencies (IC_50_ = 8.8 nM and 19 nM, respectively) with high selectivity to NSD2. Furthermore, these inhibitors exhibit prominent anti-tumor activity comparable to KRAS inhibition with sotorasib in KRAS-driven lung and pancreatic cancers, highlighting NSD2 inhibition as an emerging therapeutic strategy for aggressive solid tumors beyond hematologic malignancies [173]. Meanwhile, the covalent inhibitor BT5 targeting NSD1 has been shown to reduce H3K36me2 levels and suppress proliferation in NUP98-NSD1 fusion-driven AML models, providing proof-of-concept that direct catalytic inhibition of NSD1 is therapeutically feasible [174]. In the case of NSD3, several compounds target both NSD2 and NSD3. While LEM-14 demonstrated greater selectivity for NSD2 over NSD1 and NSD3, its derivative LEM-14-1189 showed stronger inhibitory activity against NSD3 than NSD2 (IC_50_ = 60 μM vs. 111 μM) [167]. A structure-based virtual screen identified a series of NSD-targeting inhibitors, among which compound **3** exhibited potent activities toward both NSD2 and NSD3 (IC_50_ = 0.81 μM and 0.84 μM, respectively), with anti-proliferative effects in non-small-cell lung cancer cells [175]. A recent study identified a novel inhibitor of NSD3-SET, compound **13i**, with an in vitro IC_50_ of 287 μM; despite its relatively weak biochemical potency, it suppresses proliferation of JIMT-1 breast cancer cells with a GI_50_ of 36.5 μM [176].

Collectively, these studies suggest that selective inhibition of NSD family-mediated H3K36me2 may be valuable for targeting tumors harboring overexpression or hyperactivity of NSD proteins. Nonetheless, the high sequence conservation among SET domains continues to pose challenges for achieving selectivity and minimizing off-target effects, emphasizing the need for further structural optimization and rational drug design.

### 6.2. Other Types of NSD Family Inhibitors

In addition to catalytic SET domain inhibitors, alternative approaches such as targeted protein degraders and reader domain antagonists have recently emerged as promising strategies against NSD family members (Table 2).

PROTAC-based degraders have shown particularly encouraging results. The VHL-recruiting PROTAC MS9715 selectively degraded NSD3 in breast cancer models, leading to suppression of c-Myc expression and inhibition of tumor progression [177]. Another NSD3 degrader, compound **8**, demonstrated potent antitumor activity in lung cancer models, where NSD3 degradation led to decreased expression of CDC25A and cyclin D1, induction of cell cycle arrest, and apoptosis, ultimately reducing tumor growth in xenograft models [178]. For NSD2, a protein-degradation strategy utilizing the recruitment of FBXO22 was shown to potently degrade NSD2 gain-of-function variants in ALL cells, resulting in marked growth inhibition and induction of apoptosis [179]. The first-in-class NSD2 PROTAC MS159 exhibits anti-proliferative effects in several multiple myeloma cells but lacks strict selectivity for NSD2 as it co-degrades IKZF1/3 via CRBN [180]. By contrast, UNC8153 and LLC0424 are potent, NSD2-selective degraders that reduce H3K36me2 and demonstrate anti-tumor efficacy in multiple myeloma and ALL models, respectively [181,182]. In prostate cancer, a dual NSD1/NSD2 PROTAC LLC0150 has been reported as a strategy to inhibit AR/FOXA1-driven tumorigenesis [114].

Beyond degraders, efforts have also targeted reader domains within NSD proteins. The PWWP domain of NSD3 has been validated as a therapeutic vulnerability, with the small-molecule BI-9321 selectively engaging NSD3-PWWP1 and suppressing proliferation of AML cells [183]. Similar strategies are being actively explored for NSD2-PWWP1 [184,185,186,187]. For instance, the small-molecule antagonist 3f binds the N-terminal PWWP domain of NSD2 (*K*_d_ = 3.4 μM) and blocks the interaction between NSD2 and H3K36me2, thereby disrupting NSD2-chromatin association and attenuating multiple myeloma cell growth [184]. The chemical probe UNC6934 targets NSD2-PWWP1, competitively blocks binding to nucleosomal H3K36me2, and drives aberrant relocalization of NSD2 to the nucleolus, phenocopying PWWP1-defient NSD2 isoforms [185]. In addition, compound **38** is a potent and selective NSD2-PWWP1 inhibitor that shows anti-proliferative effect in KMS-11 multiple myeloma cells [186]; further structure-based campaigns have identified additional high-affinity NSD2-PWWP1 ligands suitable for lead optimization [187]. These findings highlight the potential of developing reader domain inhibitors as an orthogonal approach to block oncogenic NSD activity.

Together, these advances underscore the feasibility of diversifying NSD-targeted therapeutic modalities beyond direct catalytic inhibition, opening new avenues for subtype-selective and context-specific cancer therapies.

**Table 2:** Inhibitors targeting NSD family members.

Types	Drugs	Targets	Inhibitory Efficacy	In Vitro/In Vivo Anti-Tumor Efficacy	Refs.
Catalytic SET inhibitor	BT5	NSD1	IC_50_ = 5.8 ± 1.4 μM (NSD1), IC_50_ = 26.7 ± 7.1 μM (NSD2), IC_50_ = 14.3 ± 6.1 μM (NSD3), (4 h incubation)	Anti-proliferative effect in NUP98-NSD1 leukemia cell line (GI_50_ = 1.3 μM at day 3) and primary samples from patients with NUP98-NSD1 fusion	[174]
	LEM-06	NSD2	IC_50_ = 0.8 mM		[166]
	DA-3003-1 PF-03882845 Chaetocin TC LPA54 ABT-199	NSD2 (no selectivity to NSD2)	IC_50_ = 0.17 μM (DA3003-1) IC_50_ = 7.6 μM (PF-03882845) IC_50_ = 0.13 μM (Chaetocin) IC_50_ = 8.5 μM (TC LPA54) IC_50_ = 1.7 μM (ABT-199)		[168]
	LEM-14	NSD2	IC_50_ = 132 μM		[167]
	LEM-14-1189	NSD1/2/3	IC_50_ = 418 μM (NSD1), 111 μM (NSD2), and 60 μM (NSD3)		[167]
	Compound **15a** (structural modification of DA-3003-1)	NSD2	IC_50_ = 0.23 μM	Apoptosis induction in cells; Anti-tumor effects in tumor xenograft (KMS-11 multiple myeloma)	[169]
	Compound **3**	NSD2, NSD3	IC_50_ = 0.81 μM (NSD2) and 0.84 μM (NSD3)	Anti-proliferative effect in vitro (non-small cell lung cancer cells)	[175]
	Compound **42**	NSD2	IC_50_ = 0.017 μM	Apoptosis induction in vitro; Anti-tumor effects in xenograft model (RS4;11 ALL cells)	[170]
	KTX-1001	NSD2	IC_50_ = 0.460 nM	Phase I clinical trial in multiple myeloma	[171,172]
	IACS-17596 IACS-17817	NSD2	IC_50_ = 8.8 nM IC_50_ = 19 nM	Clinical-grade; Anti-tumor effects in cells, xenograft, and PDX models (KRAS-driven lung and pancreatic cancers)	[173]
	13i	NSD3	IC_50_ = 287 μM	Anti-proliferative effect in JIMT-1 breast cancer cells	[176]
PWWP inhibitor	3f	NSD2	IC_50_ = 17.3 μM		[184]
	UNC6934	NSD2	IC_50_ = 104 nM	Anti-proliferative effects in t(4;14) multiple myeloma cells	[185]
	Compound **38**	NSD2	IC_50_ = 0.11 μM	Anti-proliferative effects in vitro (KMS11 multiple myeloma, ALL cells)	[186]
	Compound **34**	NSD2	pIC_50_ = 8.2 (IC_50_ ≈ 6 nM)		[187]
	BI-9321	NSD3	IC_50_ = 0.2 μM	Anti-proliferative effects in vitro (MOML-13, RN-2 AML cells)	[183]
PROTAC degrader	MS159 (E3 ligase: CRBN)	NSD2	DC_50_ = 5.2 ± 0.9 μM	Anti-proliferative effects in vitro (KMS11 and H929 multiple myeloma)	[180]
	UNC8732 (E3 ligase: FBXO22)	NSD2	DC_50_ = 60 nM	Anti-proliferative effects in vitro (NSD2 E1099K ALL cells)	[179]
	UNC8153 (E3 ligase: FBXO22)	NSD2	IC_50_ = 0.35 μM	Anti-proliferative effects in multiple myeloma cells	[181]
	LLC0424 (E3 ligase: CRBN)	NSD2	DC_50_ = 20 nM	Anti-proliferative effects in vitro (ALL cells); NSD2 degradation in vivo	[182]
	MS9715 (E3 ligase: VHL)	NSD3	DC_50_ = 4.9 ± 0.4 μM	Anti-proliferative effects in vitro (MLL-rearranged AML and multiple myeloma cells)	[177]
	Compound **8** (E3 ligase: VHL)	NSD3	DC_50_ = 0.94 ~ 1.43 μM (reduces H3K36me2)	Induces apoptosis and cell cycle arrest in lung cancer cells	[178]
	LLC0150	NSD1/2	IC_50_ = 0.274 ~ 69.68 nM	Anti-proliferative effects in vitro and in vivo (prostate cancer)	[114]

### 6.3. Indirect Inhibition of NSD Family Function

In malignancies where NSD family proteins are key oncogenic drivers, such as NUT carcinoma and LUSC, BET inhibition has emerged as a promising therapeutic strategy. Indirect strategies for suppressing NSD family-driven oncogenesis frequently involve modulation of bromodomain protein BRD4 and its chromatin interaction partners. BRD4 interacts with NSD3-short, serving as an adaptor to facilitate oncogenic transcription in multiple cancers, including NUT carcinoma and NSD3-NUT fusion-positive LUSC [26].

In NUT carcinoma featuring NSD3-NUT gene fusions, NSD3 interacts with BRD4, forming complexes that block cellular differentiation and sustain tumor growth. BET inhibitor treatment disrupts this NSD3-BRD4 complex, restores differentiation, and effectively suppresses tumor cell proliferation [50]. In murine LUSC models with either NSD3 amplification or the activating NSD3(T1232A) mutation, tumor growth is notably diminished following BET inhibitor administration. Both cell line models and patient-derived xenograft (PDX) studies indicate that NSD3 dependency is predictive of BET inhibitor sensitivity [23].

Other indirect modulators of NSD family function are being explored as promising adjuncts for cancer therapy. ATRA has emerged as a promising agent for multiple myeloma cases characterized by t(4;14) translocation and NSD2 overexpression. Mechanistically, ATRA enhances CD38 expression by stabilizing RARα and increasing H3K36me2 enrichment at the CD38 promoter, thus providing a rationale for its application to potentiate CD38-targeted immunotherapies [158]. In TNBC, NSD2 is overexpressed and promotes tumor growth through EGFR–AKT signaling. NSD2 depletion enhances sensitivity to the EGFR inhibitor gefitinib, suggesting that NSD2 indirectly regulates drug resistance via its methyltransferase activity and may serve as a combinatorial target in TNBC therapy [188]. Resistance to proteasome inhibition in MM, particularly bortezomib, has been linked to the interaction between NSD2 and SRC3. NSD2-mediated stabilization of SRC3 and augmented liquid–liquid phase separation leads to enhanced H3K36me2 modification on anti-apoptotic gene promoters, contributing to drug resistance. Disrupting the NSD2–SRC-3 interaction with novel inhibitors like SI-2 sensitizes MM cells to bortezomib and helps overcome resistance, unveiling an epigenetic mechanism underlying chemoresponsiveness [157].

Together, these approaches underscore the value of indirect NSD modulation for overcoming drug resistance and achieving targeted anti-cancer effects in diverse hematologic and solid malignancies.

### 6.4. Potential Toxicity and Safety Consideration of NSD Inhibition

Pharmacological inhibition of the NSD family of histone methyltransferases repre-sents a promising anticancer strategy; however, potential toxicity remains a major concern. NSD1, NSD2, and NSD3 play crucial roles in normal chromatin organization, DNA damage repair, and developmental gene regulation, so systemic inhibition risks disrupting physiological epigenetic homeostasis.

Preclinical studies have shown that broad depletion of H3K36me2 is associated with transcriptional stress, impaired cellular differentiation, and genomic instability in non-malignant cells [173]. Inhibition of NSD2 or NSD3, especially via SET domain or PWWP domain inhibitors, can affect hematopoietic and neuronal gene expression patterns, raising concerns about possible hematologic and neurodevelopmental adverse effects [1,23,186].

Moreover, catalytic inhibitors competing at the SAM-binding pocket may have off-target impacts on other SET-domain methyltransferases, such as SETD2 or EZH2, potentially leading to unintended epigenetic alterations [1,174]. Thus, achieving isoform-specific selectivity, tumor-targeted drug delivery, and reversible modulation of NSD enzymatic activity are critical goals to minimize toxicity risks during clinical development.

Future research should emphasize comprehensive toxicogenomic and pharmacoki-netic profiling to delineate safe therapeutic windows and ensure on-target specificity and safety of NSD inhibitors.

## 7. Conclusions and Perspectives

The NSD family of histone methyltransferases has emerged as a central epigenetic regulator with multifaceted roles during cancer development. Through both H3K36 methylation-dependent and -independent mechanisms, NSD enzymes contribute to multiple hallmarks of cancer, including malignant transformation, uncontrolled proliferation, invasion, metastasis, metabolic rewiring, immune evasion, and therapeutic resistance. Moreover, the existence of both catalytic and non-catalytic isoforms further diversifies their oncogenic potential across hematologic and solid tumors. Accordingly, cancer-associated genetic alterations in NSD family members may serve as valuable biomarkers for prognosis and treatment response, and isoform-specific targeting has become a promising strategy for treatment of cancers driven by NSD dysregulation.

Despite major advances in defining the functional roles of the NSD family and in developing selective inhibitors, several challenges remain. First, the context-dependent dual roles of NSDs as either an oncogene or tumor suppressor remain incompletely defined. Mechanistic dissection of cofactor recruitment, chromatin state, mutation class, and lineage-specific transcriptional dependencies will be necessary to elucidate the molecular basis of this bidirectional activity. Second, although several studies have limited NSDs to tumor-infiltrating immune cell signatures, their precise roles within the tumor microenvironment remain poorly characterized and, in some cases, controversial. In addition, their contribution to resistance to targeted therapies or immunotherapies is insufficiently understood. Elucidating tumor-extrinsic functions of NSD family members beyond tumor-intrinsic mechanisms may provide novel rationales for combinatorial therapeutic approaches. Third, the development of NSD-targeted therapeutics is still challenged by substrate competition, enzymatic redundancy, high structural similarity within SET domain, and potential for systemic toxicity. Structure-guided design of subtype-selective inhibitors, proteolysis-targeting degraders, and rational combination strategies may help overcome these limitations.

Taken together, the NSD family represents both a mechanistic cornerstone of cancer epigenetics and a dynamic frontier for therapeutic intervention. Continued integration of structural, functional, and translational research is poised to accelerate the development of selective and clinically effective NSD-targeted therapies, ultimately improving outcomes for patients with NSD-driven malignancies.

## Data Availability

No new data were created or analyzed in this study. Data sharing is not applicable to this article.

## Abbreviations

The following abbreviations are used in this manuscript:

AK2	Adenylate kinase 2
AML	Acute myeloid leukemia
ALL	Acute lymphoblastic leukemia
AR	Androgen receptor
AROS	Active regulator of SIRT1
ASH1L	ASH1-like
ATRA	All-trans retinoic acid
BET	Bromodomain and extraterminal
CN	Cytogenetically normal
DDR	DNA damage response
DSB	Double-strand break
EMT	Epithelial–mesenchymal transition
ERα	Estrogen receptor alpha
G6PD	Glucose-6-phosphate dehydrogenase
HCC	Hepatocellular carcinoma
HGSC	High-grade serous carcinoma
HK2	Hexokinase 2
H3K36	Histone lysine 36
H3K36me1	Histone lysine 36 monomethylation
H3K36me2	Histone lysine 36 dimethylation
H3K36me3	Histone lysine 36 trimethylation
HMG	High mobility group
HNSCC	Head and neck squamous cell carcinoma
HR	Homologous recombination
IRF3	Interferon regulatory factor 3
JHDM	Jumonji C (JmjC) domain-containing histone demethylase
LCA	Laryngeal cancer
LUAD	Lung adenocarcinoma
LUSC	Lung squamous cell carcinoma
MLL1	Mixed lineage leukemia 1
NHEJ	Non-homologous end joining
NSL	Non-specific lethal
NUP98	Nucleoporin 98
NSD	Nuclear receptor-binding SET domain-containing protein
NSRP1	Nuclear speckle splicing regulatory protein 1
PHD	Plant homeodomain
PROTAC	PROteolysis-Targeting Chimera
PWWP	Proline-tryptophan-tryptophan-proline
SAM	S-adenosylmethionine
SET	Su(var)3-9, enhancer of zeste and trithorax
SETMAR	SET domain and mariner transposase fusion gene-containing
SMYD2	SET and MYND domain-containing 2
TCGA	The Cancer Genome Atlas
TMB	Tumor mutation burden
TNBC	Triple-negative breast cancer
VEGF-A	Vascular endothelial growth factor-A
